# Mechanism of Action of *Fusarium oxysporum* CCS043 Utilizing Allelochemicals for Rhizosphere Colonization and Enhanced Infection Activity in *Rehmannia glutinosa*

**DOI:** 10.3390/plants14010038

**Published:** 2024-12-26

**Authors:** Feiyue Yuan, Fuxiang Qiu, Jiawei Xie, Yongxi Fan, Bao Zhang, Tingting Zhang, Zhongyi Zhang, Li Gu, Mingjie Li

**Affiliations:** 1 College of Bee Science and Biomedicine, Fujian Agriculture and Forestry University, Fuzhou 350002, China; yfy1829@126.com (F.Y.); fuxiangqiu010@163.com (F.Q.); zyzhang@fafu.edu.cn (Z.Z.); 2 Key Laboratory of Ministry of Education for Genetics, Breeding and Multiple Utilization of Crops, Fujian Agriculture and Forestry University, Fuzhou 350002, China; 13705063268@163.com; 3 College of JunCao Science and Ecology, Fujian Agriculture and Forestry University, Fuzhou 350002, China; fyx18005096100@163.com; 4 College of Bioengineering, Henan University of Technology, Zhengzhou 450001, China; baozhang923@163.com

**Keywords:** *Rehmannia glutinosa*, *F. oxysporum* CCS043, allelopathy, root exudates, effector protein

## Abstract

*Rehmannia glutinosa* is an important medicinal herb; but its long-term cultivation often leads to continuous cropping problems. The underlying cause can be attributed to the accumulation of and alterations in root exudates; which interact with soil-borne pathogens; particularly *Fusarium oxysporum*; triggering disease outbreaks that severely affect its yield and quality. It is therefore crucial to elucidate the mechanisms by which root exudates induce *F. oxysporum* CCS043 outbreaks. In this study; the genome of *F. oxysporum* CCS043 from *R. glutinosa’s* rhizosphere microbiota was sequenced and assembled de novo; resulting in a 47.67 Mb genome comprising 16,423 protein-coding genes. Evolutionary analysis suggests that different *F. oxysporum* strains may adapt to the host rhizosphere microecosystem by acquiring varying numbers of specific genes while maintaining a constant number of core genes.The allelopathic effects of ferulic acid; verbascoside; and catalpol on *F. oxysporum* CCS043 were examined at the physiological and transcriptomic levels. The application of ferulic acid was observed to primarily facilitate the proliferation and growth of *F. oxysporum* CCS043; whereas verbascoside notably enhanced the biosynthesis of infection-related enzymes such as pectinase and cellulase. Catalpol demonstrated a moderate level of allelopathic effects in comparison to the other two. Furthermore; 10 effectors were identified by combining the genomic data. Meanwhile; it was found that among the effector-protein-coding genes; ChiC; VRDA; csn; and chitinase exhibited upregulated expression across all treatments. The expression patterns of these key genes were validated using qRT-PCR. Transient overexpression of the two effector-encoding genes in detached *R. glutinosa* leaves provided further confirmation that ChiC (GME8876_g) and csn (GME9251_g) are key effector proteins responsible for the induction of hypersensitive reactions in *R. glutinosa* leaf cells. This study provides a preliminary indication that the use of allelochemicals by *F. oxysporum* CCS043 can promote its own growth and proliferation and enhance infection activity. This finding offers a solid theoretical basis and data support for elucidating the fundamental causes of fungal disease outbreaks in continuous cropping of *R. glutinosa* and for formulating effective mitigation strategies.

## 1. Introduction

*Rehmannia glutinosa* (*R. glutinosa*) is one of the most frequently used herbs in traditional Chinese medicine (TCM), with the history of its usage spanning thousands of years in China [1]. However, during the cultivation process, the severe problem of obstacles to continuous cropping is encountered, which requires the same land to be left fallow for a period of 8–10 years following the harvesting a crop of *R. glutinosa* [2,3]. Statistical evidence has shown that over 70% cases of continuous cropping problems are accompanied by the occurrence of soil-borne diseases, a phenomenon that is particularly pronounced in the production of root- or rhizome-based medicinal materials. In their natural environments, the healthy growth and biomass accumulation of plants are largely dependent on a balanced relationship between the plant and the rhizosphere’s microbial community [4,5].

*Fusarium oxysporum* CCS043, a pathogenic fungus, has a pervasive presence in the soil surrounding plant roots [6]. This fungus is frequently found in medicinal plants, where it causes significant problems with continuous cropping. Its colony numbers and relative abundance are significantly increased under continuous cropping conditions, and it occupies more ecological niches within the microbial community [7]. Studies have shown that the gradual synthesis and accumulation of root exudates during the growth of *R. glutinosa* are considered to be the primary causes of continuous cropping problems in *R. glutinosa* [8,9]. The allelochemicals identified as key components include ferulic acid, verbascoside, and catalpol [10,11]. Furthermore, other studies have suggested that the formation of continuous cropping problems is thought to involve interactions among plants, allelochemicals, and microorganisms within the rhizosphere microecosystem [12,13,14,15].

It is worthy of note that an innate immune system has been developed by plants through their long-term co-evolution with pathogenic microorganisms. This system includes two lines of defense, namely pattern-triggered immunity (PTI) and effector-triggered immunity (ETI) [16,17,18]. The activation of a plant’s ETI occurs when resistance genes recognize the effector proteins produced by pathogens, which are substances secreted by Gram-negative bacteria, fungi, and oomycetes into plant cells that can suppress their PTI responses. These genes are typically recognized by intracellular NBS-LRR or cell surface RLK/RLP receptors, which often induce a robust immune response and lead to host cell death [19]. Consequently, an understanding of the interaction between allelochemicals and pathogenic microorganisms is vital for elucidating the causes of obstacles to continuous cropping.

The prevailing view among researchers is that the outbreak of fungal diseases mediated by root exudates, especially *F. oxysporum* CCS043, is considered to represent a core characteristic of continuous cropping obstacles in *R. glutinosa*. Despite the mechanisms underlying this process remaining unclear, this remains the dominant hypothesis [2,9,19]. In this study, potential autotoxic allelochemicals were exogenously added to simulate continuous cropping conditions, and their allelopathic effects on *F. oxysporum* CCS043 were evaluated. Effector proteins were identified based on genome and transcriptome data for *F. oxysporum* CCS043 in the rhizosphere of *R. glutinosa*, and the evolutionary relationships of *F. oxysporum* CCS043, as well as key response pathways to potential allelochemicals, were explored. Additionally, transient overexpression of candidate effectors in detached *R. glutinosa* leaves was induced to identify the key effectors involved in the infection process. This study provides critical information on the key effectors of *F. oxysporum* CCS043 in the rhizosphere of *R. glutinosa*, offering an important theoretical reference for elucidating the mechanisms of fungal disease outbreaks mediated by root exudates.

## 2. Results

### 2.1. Whole-Genome Sequencing, De Novo Assembly, and Annotation of F. oxysporum CCS043 from the Rhizosphere of R. glutinosa

The evolutionary process of *F. oxysporum* CCS043 in the rhizosphere of *R. glutinosa* was investigated through whole-genome sequencing. The highest peak of the 15-mer frequency of the Illumina short reads was observed at a depth of 28 (Figure S1). The estimated genome size of *F. oxysporum* CCS043 was determined to be 61.05 Mb, with the actual assembled genome size being 47.67 Mb. The DNBSEQ platform was employed for whole-genome sequencing, resulting in a total of 20.46 Gb (429X coverage). The genome was found to consist of 45 scaffolds, with a scaffold N50 value of 4451.03 Kb. The homology, de novo gene structure, and transcriptome-assisted prediction revealed that *F. oxysporum* CCS043 has 16,423 genes encoding proteins, with an average gene length and coding sequence (CD) of 1615.58 bp and 1477.40 bp, respectively (Table 1). Gene annotation of *F. oxysporum* CCS043 was carried out using 15 databases to ascertain the gene information. The NR database yielded the highest number of annotated genes (16,211), while the CARD database yielded the fewest (1) (see Table S1 for details) (Table S1). The five major types of repeats in the genome (DNA transposons, LINEs, LTRs, SINEs, and unknown repeats) were further identified through de novo methods. The largest proportion of the genome was found to be occupied by unknown repeat sequences (39.17%; total length: 984.55 Kb), followed by LTRs (37.73%; total length: 940.93 Kb), DNA transposons (13.86%), LINEs (9.62%), and SINEs (0.122%) (Table S2). Furthermore, 562 non-coding RNAs (ncRNAs) were predicted in the genome, comprising 68 rRNAs, 104 miRNAs, 305 tRNAs, 48 sRNAs, and 37 snRNAs (Table S3).

### 2.2. Evolution of F. oxysporum CCS043 in the Rhizosphere of R. glutinosa

**Figure 1:**
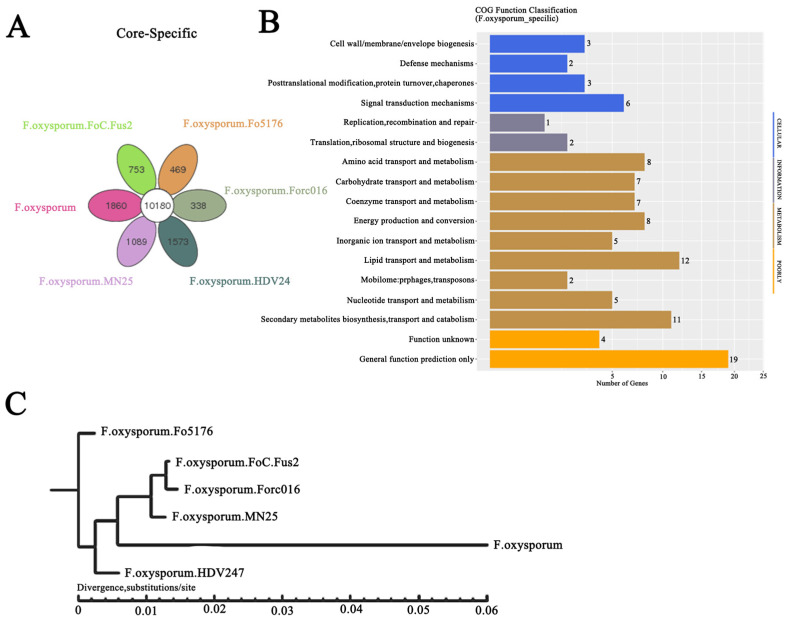
Analysis of gene loss and unique genes in *F. oxysporum* CCS043 from the rhizosphere of *R. glutinosa*. Panel (**A**) is a Venn diagram showing the core and unique genes shared among six strains of *F. oxysporum* CCS043; panel (**B**) is a COG functional prediction chart for the 1860 unique genes from *F. oxysporum* CCS043 from the rhizosphere of *R. glutinosa*; panel (**C**) is a phylogenetic tree constructed using the common protein sequences of the six *F. oxysporum* strains.

After conducting a comparative analysis of the genome of *F. oxysporum* CCS043 from the rhizosphere of *R. glutinosa* and those of *F. oxysporum* from other crops, it was found that *F. oxysporum* CCS043 from the rhizosphere of *R. glutinosa* had the fewest genes, totaling 16,423 (Table S4). However, in terms of unique genes, it had the highest number among the six strains, with 1860 unique genes (Table S5 and Figure 1A). This suggests that different *F. oxysporum* strains may acquire varying numbers of specific genes without losing their core genes during the process of adapting to their hosts. Additionally, an evolutionary tree was constructed, and COG functional annotation was performed based on the core and unique protein sequences, which showed that among the core genes, *F. oxysporum* CCS043 from the rhizosphere of *R. glutinosa* was most closely related to *F. oxysporum*. FoC.Fus2, *F. oxysporum*. Forc016, and *F. oxysporum*. MN25, as depicted in Figure 1C. The unique genes in *F. oxysporum* CCS043 from the rhizosphere of *R. glutinosa* were significantly enriched in cellular components, including those related to signal transduction, the cell membrane, and the cell wall. Additionally, the metabolic pathways were significantly enriched in lipid metabolism, synthesis, transport, and the catabolism of secondary metabolites, as illustrated in Figure 1B. Based on these findings, it can be inferred that *F. oxysporum* CCS043 from the rhizosphere of *R. glutinosa* has undergone genomic evolution, which facilitates its adaptation to the host environment.

### 2.3. The Allelopathic Effects of Key Components of Root Exudates from Continuous Cropping of R. glutinosa on F. oxysporum CCS043

**Figure 2:**
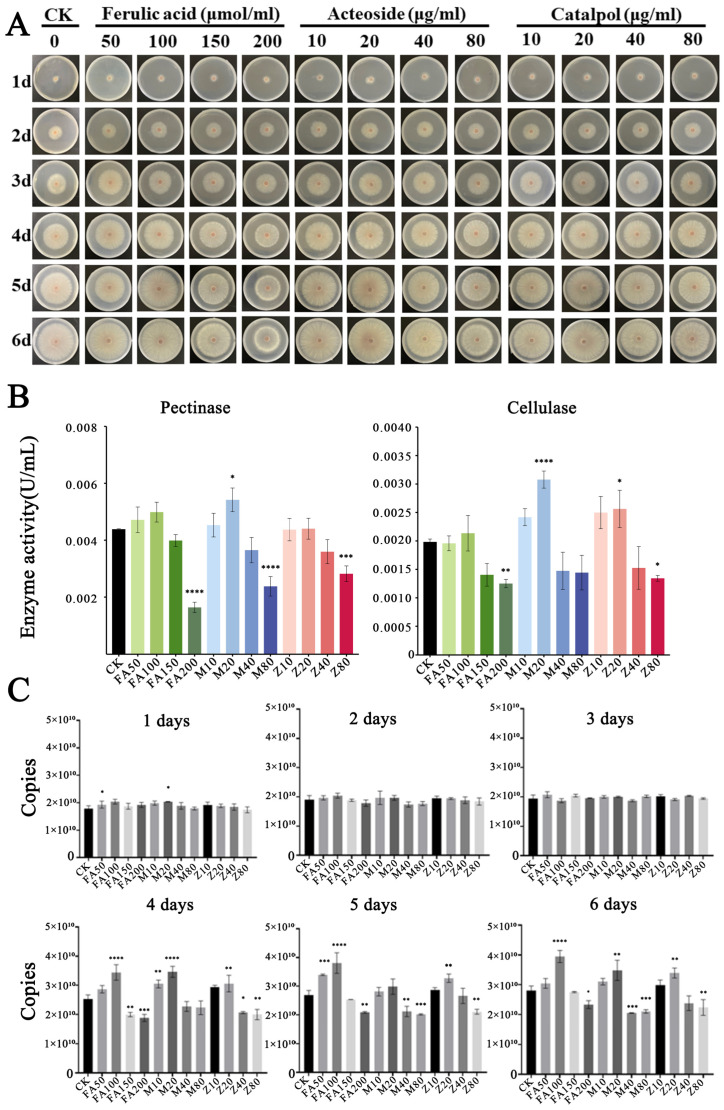
Physiological changes in *F. oxysporum* CCS043 upon the addition of key components from the root exudates of *R. glutinosa*. Panel (**A**) indicates the growth of *F. oxysporum* CCS043 with the addition of ferulic acid, verbascose, and catalpol. Panel (**B**) depicts the activities on the left and right of pectinase and cellulase in fermentation broth of *F. oxysporum* CCS043, respectively. Panel (**C**) illustrates the changes in the abundance of *F. oxysporum* CCS043 under treatments with ferulic acid, verbascose, and catalpol, where “d” refers to days, and the treatment names with “FA” stand for ferulic acid, with concentration units in umol/L; “M” refers to verbascose, with concentration units in ug/mL; and “Z” refers to catalpol, with concentration units in ug/mL. The symbol “*” indicates significant differences between data points (*p* < 0.05), “**” indicates significant differences between data points (0.01 < *p* < 0.05), “***” indicates significant differences between data points (*p* < 0.001), and “****” indicates significant differences between data points (*p* < 0.0001).

Following the addition of the root exudates of *R. glutinosa* for a period of 6 days, the diameter of the fungal colony was measured (Figure 2A and Figure S2). A significant trend of hyphal proliferation was observed when the concentrations of ferulic acid, verbascose, and catalpol were set at 100 µmol/L, 20 µg/mL, and 20 µg/mL, respectively, with the growth rates reaching 21.15%, 6.69%, and 9.04%. However, when the concentrations were increased to 200 µmol/L, 80 µg/mL, and 80 µg/mL, a notable inhibitory effect was observed, with inhibition rates of 38.2%, 16.3%, and 25.3%, respectively. These findings indicate that ferulic acid, verbascose, and catalpol exert a significant influence on the proliferation and hyphal growth of *F. oxysporum* CCS043, exhibiting a pattern where lower concentrations promote growth and higher concentrations suppress it (Figure 2A). Concurrently, as the hyphae developed, changes in the activities of pectinase and cellulase in *F. oxysporum* CCS043 were also observed. At a verbascose concentration of 20 μg/mL, pectinase activity was significantly enhanced. Similarly, cellulase activity in *F. oxysporum* CCS043 was highly significantly increased at the same verbascose concentration, reaching its peak on the third day. This enhancement in pectinase and cellulase activities plays a role in the rapid degradation of the host cell wall, thereby facilitating the infection of *R. glutinosa* by *F. oxysporum* CCS043 (Figure 2B). Furthermore, daily sampling from the plates shown in Figure 2A was carried out to assess the colony abundance (Figure 2C). It was determined that during the initial three days, the abundance of *F. oxysporum* CCS043 in both the control (CK) and treatment groups exhibited a gradual increase, with no significant difference observed. However, on the fourth day post-inoculation, with the addition of ferulic acid, verbascose, and catalpol at concentrations of 100 μmol/L, 20 μg/mL, and 20 μg/mL, respectively, the abundance of *F. oxysporum* CCS043 was 1.33 times, 1.28 times, and 1.10 times higher than that in the CK, achieving a significantly higher abundance than the CK. Although the abundance on the fifth and sixth days remained higher than that in the CK, the increase was not as pronounced as that on the fourth day. This suggests that the fourth day represents a critical phase for the abundance of *F. oxysporum* CCS043, as an exceptionally active growth phase for the fungus.

### 2.4. Analysis of Gene Differential Expression Patterns and Metabolic Pathway Enrichment in F. oxysporum CCS043

**Figure 3:**
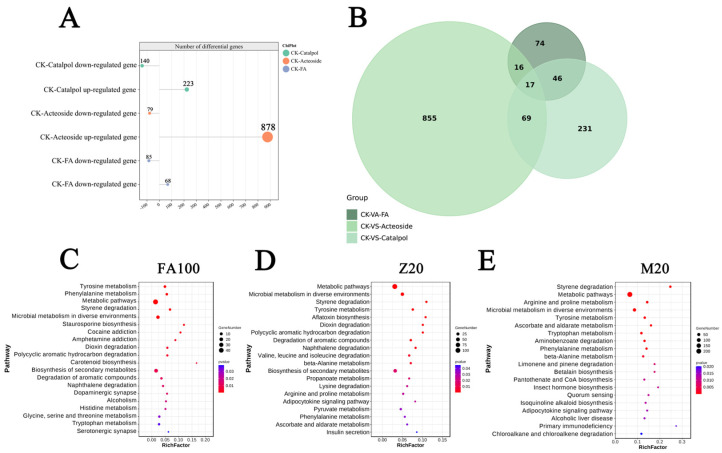
Number of differentially expressed genes and KEGG enrichment. (**A**) presents a bar chart displaying the quantity of differentially expressed genes; (**B**) depicts a Venn diagram that illustrates the differentially expressed genes under the influence of the three distinct additives; (**C**–**E**) show scatter plots representing the KEGG enrichment of the differentially expressed genes among the ferulic acid, acteoside, and catalpol treatment groups, respectively. In the figure, a higher Richfactor signifies a greater extent of enrichment; the color of the points approaching red indicates a smaller *p*-value; and the size of the points corresponds to the number of differentially expressed genes, with larger points denoting a greater quantity of genes.

To clarify the molecular mechanisms through which *F. oxysporum* CCS043 employs autotoxic substances derived from the continuous cropping of *R. glutinosa* to proliferate and enhance its infectivity, the samples were cultured in media supplemented with 100 μmol/L ferulic acid (FA100), 20 μg/mL acteoside (M20), and 20 μg/mL catalpol (Z20) and a control medium lacking the three allelochemical components (CK) until the fourth day. The sequencing results indicated that the Q30 values for the 12 groups of data ranged from 88.75 to 90.20, while the Q20 values were all above 95.88%, thereby signifying that the databases obtained had high-quality original reads (Table S6). Furthermore, employing a screening threshold of |log2Fold Change| ≥ 1 and a *p*-value ≤ 0.05, it was found that the ferulic acid treatment group had a total of 153 differentially expressed genes. Of these, 68 were found to be upregulated, and 85 were found to be downregulated. The catalpol treatment group displayed 363 differentially expressed genes, comprising 223 upregulated and 140 downregulated genes, while 17 differentially expressed genes were common to all three treatments (Figure 3A,B). Subsequently, a KEGG enrichment analysis was then performed on the differentially expressed genes from these three groups (Figure 3C–E). Notably, the metabolic pathways were found to be highly similar across the three groups, with pathways such as metabolic pathways, tyrosine metabolism, and styrene degradation being significantly enriched in all three groups. This suggests that ferulic acid, acteoside, and catalpol may exert similar effects on *F. oxysporum* CCS043 in the rhizosphere of *R. glutinosa* through analogous mechanisms, thereby facilitating the massive proliferation of the fungus and providing substantial reinforcements for attacking the root system of *R. glutinosa*.

### 2.5. Screening and Identification of Effector Proteins in F. oxysporum CCS043 from the Rhizosphere of R. glutinosa

Screening of the effector proteins was conducted bioinformatically from the 16,423 genes encoding proteins obtained through whole-genome sequencing. This was based on the identification of conserved characteristics, including the presence of secretion signal peptides, the absence of transmembrane domains, a smaller molecular weight, and a high content of cysteine residues. SignalP v5.0 was used to predict the signal peptides, resulting in the identification of 1542 proteins with these peptides. A further analysis of the transmembrane domains revealed that 1286 proteins lacked such domains and were retained (Figure S3). Considering the small-molecular-weight feature of effector proteins, proteins with amino acid residues spanning the range of 50 to 300 were identified, resulting in the exclusion of one protein with less than 50 aa and 801 proteins with more than 300 aa. This process yielded 484 proteins (Table S7). ProtComp v.9.0 was then used to perform a localization analysis on the 484 candidate proteins, indicating that 414 proteins were secreted into the extracellular space and distributed among various cellular components, with the highest number in the mitochondria (32) and the lowest in the vacuoles (1) (Table S8). Big-PIPredictor was employed for GPI-anchored protein prediction, resulting in the screening of 56 GPI-anchored proteins and 358 non-GPI-anchored proteins (Table S9). Furthermore, sequences devoid of cysteine were excluded on the basis of a high cysteine content being a defining characteristic of effector proteins. This resulted in the retention of 189 protein sequences exhibiting a cysteine residue content of 6 or more (Table S10). Finally, the 189 protein sequences with a cysteine residue content of 6 or more were compared with the PHIB database, and 10 protein sequences were annotated as candidate pathogenicity-related effectors (Table 2).

### 2.6. qRT-PCR Validation of the Key Genes in F. oxysporum CCS043’s Response to Root Exudates

**Figure 4:**
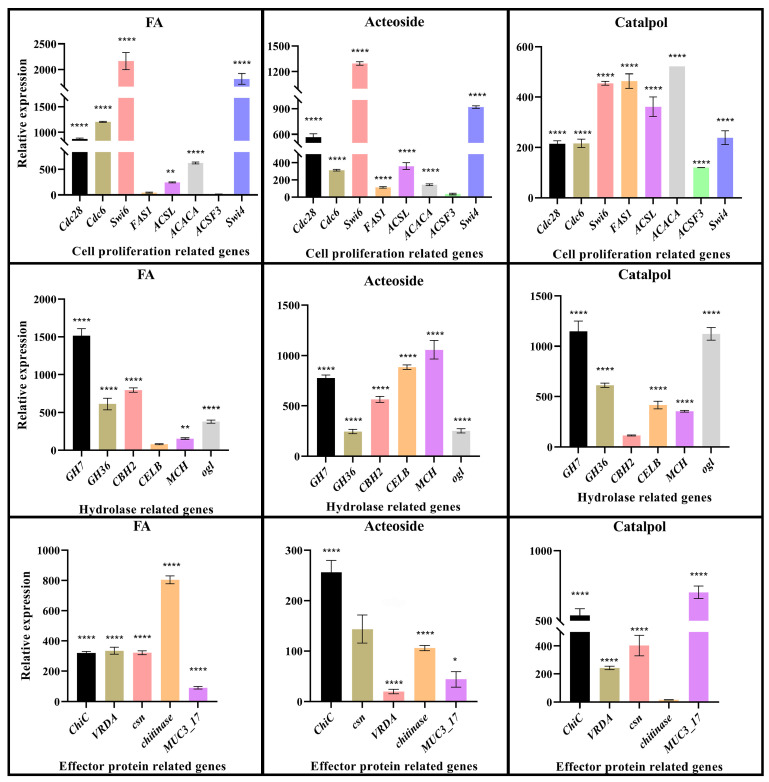
The figure indicates the relative expression levels of the genes encoded by *F. oxysporum* CCS043. In the treatment names, “FA” represents ferulic acid; “Acteoside” refers to verbascoside; and “Catalpol” represents catalpol. The symbol “*” indicates significant differences between data points (*p* < 0.05), “**” indicates significant differences between data points (0.01 < *p* < 0.05), and “****” indicates significant differences between data points (*p* < 0.0001).

To investigate the impact of root exudates from the continuous cropping of *R. glutinosa* on the transcriptional level of *F. oxysporum* CCS043 further, qRT-PCR determination was conducted on selected genes. The validated set of genes is detailed in Table S11. Consequently, regarding genes associated with cell growth (Figure 4), a markedly significant differential gene expression was noted for Swi6, Swi4, Cdc26, Cdc28, and ACACA following the treatment with ferulic acid, with upregulation that was 2164, 1817, 1201, 879.4, and 619.9 times greater, respectively, compared to the control group (CK). In the treatment group with verbascoside, the expression of Cdc28, Swi4, Swi6, Cdc26, FAS1, ACSL, and ACACA was increased by 567.1, 918.8, 1294, 313.5, 111.6, 360.7, and 142 times, respectively. In the catalpol treatment group, the expression levels of ACSF3, Swi6, Swi4, FAS1, ACSL, ACACA, Cdc28, and Cdc26 were found to be increased by 120.6, 453.9, 237.8, 462.6, 360.7, 520.8, 213.8, and 215.5 times, respectively, in comparison to the CK group. Overall, the difference in the gene expression between the ferulic acid treatment group and the CK group was extremely significant, with a clear upward trend, suggesting that in the presence of ferulic acid, genes related to cell division and growth are highly expressed, thereby enhancing the growth of *F. oxysporum* CCS043 in the rhizosphere of *R. glutinosa*.

For infection-enzyme-related genes, the ferulic acid treatment significantly upregulated *Gh7*, *GH36*, *CBH2,* and *ogl* by 1515, 610.7, 795.4, and 377.9 times, respectively, compared to the control (CK). In the verbascoside treatment group, *GH7*, *CBH2*, *CELB*, *MCH*, *GH36,* and ogl were upregulated by 777.3, 562.9, 883.6, 1056, 243.6, and 251 times, respectively, while the catalpol treatment resulted in the highest increases for *GH7*, *CBH2*, *CELB*, *MCH*, and ogl, with their expression levels rising by 1147, 111.7, 415.1, 358.4, and 1121 times, respectively. The verbascoside treatment showed the strongest overall upregulation, suggesting that it enhances infection-enzyme-related genes, thereby boosting this strain of *F. Oxysporum* CCS043’s infection ability against *R. glutinosa*.

For effector-protein-encoding genes, the ferulic acid treatment upregulated chitinase, *VRDA*, *ChiC*, and *csn* by 803.3, 334.2, 320.6, and 321.7 times, respectively. In the verbascoside group, *ChiC*, *csn,* and chitinase were upregulated by 255.3, 142.5, and 104.9 times, while in the catalpol group, *VRDA*, *csn*, *ChiC,* and *MUC3_17* were increased by 241.9, 402.1, 536.4, and 702.4 times, respectively. *Csn* and *ChiC* consistently showed significant upregulation across all of the treatments. Ferulic acid led to the highest overall upregulation of the effector protein genes, indicating its role in enhancing plant–pathogen interactions.

### 2.7. The Detection of Infection Effects of F. oxysporum CCS043’s Effector Factors on R. glutinosa

**Figure 5:**
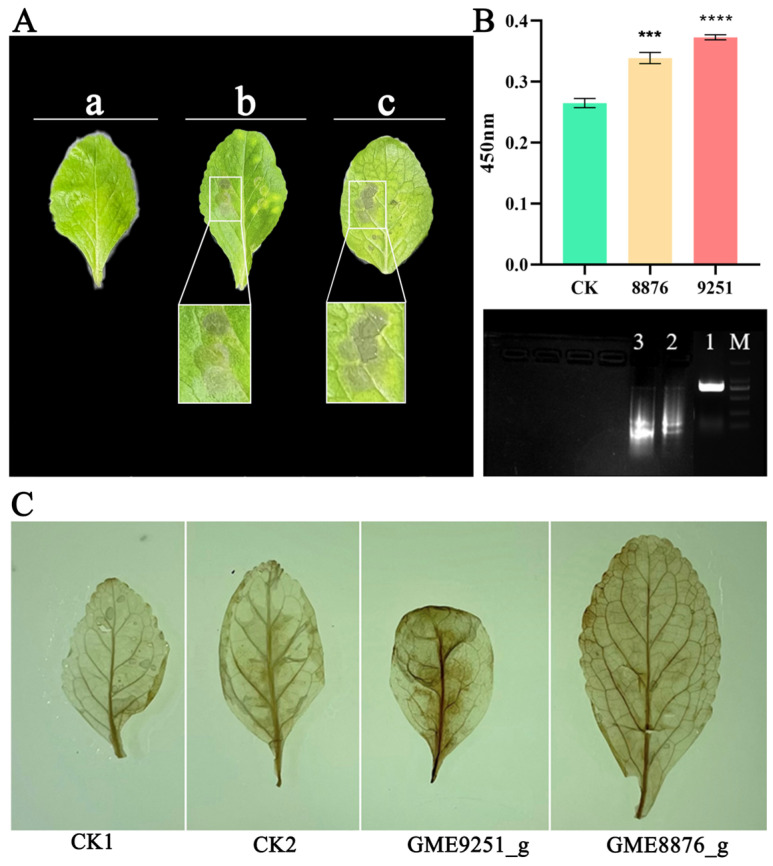
Physiological identification of effector proteins in in vitro infection. Image (**A**) indicates the candidate effector proteins infecting the *R. glutinosa* leaves in vitro; image (**B**) indicates the characterization of ROS in the *R. glutinosa* leaves infected according to the effector proteins from Image A, with DNA agarose gel electrophoresis detection below and M being the 2000 bp marker, 1 being the CK (control), 2 being the degradation band of GME8876_g, and 3 being the degradation band of GME9251_g; image (**C**) shows DAB staining of the *R. glutinosa* leaves infected in vitro, with 1–2 being the CK and 3 being *GME9251_g*. a: PBI121 empty vector; b: *GME8876_g*; c: *GME9251_g*. The symbol “***” indicates significant differences between data points (*p* < 0.001), and “****” indicates significant differences between data points (*p* < 0.0001).

To gain further insight into the functions of the effector proteins during the infection process of *F. oxysporum* CCS043 in *R. glutinosa* plants, cloning of 10 predicted effector protein genes was conducted. These were subsequently constructed into the PBI121 overexpression vector for the purpose of transiently infecting leaves of *R. glutinosa* in vitro. After 3 days of infection, the phenotypic display indicated that the leaves overexpressing the effector proteins *GME8876_g* and *GME9251_g* developed necrotic spots in comparison to the empty vector control, with those with *GME9251_g* overexpression exhibiting the most severe symptoms (Figure 5A). This suggests that hypersensitive reactions are caused by *GME8876_g* and *GME9251_g* in the leaves, leading to apoptosis in *R. glutinosa* leaf cells. Furthermore, detection of the ROS content in the detached leaves revealed that the accumulation of ROS in the detached leaves of infected *R. glutinosa* was significantly higher compared to that in the control group (Figure 5B). Meanwhile, the DAB-stained leaves in the treatment group exhibited a darker color than those in the control group, indicating a higher accumulation of H_2_O_2_ in the leaves, which confirmed the functions of these two genes further (Figure 5C). Finally, the electrophoresis results in terms of the DNA degradation bands (Figure 5B) indicated that genomic DNA degradation was evident in the experimental group following infection, as evidenced by the presence of ladder-like and diffuse, small DNA fragments. In contrast, the *R. glutinosa* leaves from the control group did not display this phenomenon. This indicates that the effector proteins GME8876_g and GME9251_g may increase the levels of ROS and H_2_O_2_ in *R. glutinosa*, thereby providing a defense against the invasion of pathogenic fungi in the rhizosphere of *R. glutinosa*.

## 3. Discussion

### 3.1. The Co-Evolutionary Relationship Between Continuous R. glutinosa Cultivation and F. oxysporum CCS043

A variety of adverse stresses and forms of ecological niche competition with other pathogens in the natural environment exert strong selective pressures on pathogens, which are forced to adapt through continuous evolution to ensure their survival. The process of host adaptation is central to the evolution of pathogens, and the interaction between hosts and pathogens is a complex process. Pathogens that originally have a broad host spectrum gradually evolve into host-adapted or host-specific pathogens through ongoing selection and evolution [20,21,22]. Furthermore, research findings indicate that host-specific pressures drive the evolution of pathogenicity and niche specialization in *F. oxysporum*. By retaining core genes while acquiring unique genes, this strain ensures both adaptability to its host and the preservation of its essential genetic functions. These findings provide a foundation for further research into the roles of specific genes in host–pathogen interactions and their potential applications in controlling host-specific pathogenic strains.

### 3.2. The Ten Effector Proteins of F. oxysporum CCS043 from the Rhizosphere of R. glutinosa Identified

The host range of *F. oxysporum* CCS043 is potentially determined to be quite narrow due to the specific recognition of effector proteins [22]. It is speculated that this may be due to the formation of new effector variants in response to effectors from other plants, facilitating the invasion of *R. glutinosa*. The rice blast fungus exhibits a variety of strains, with each displaying distinct variants of the Avr-Pik effector protein. These variations are primarily driven by the need to adapt to different hosts, thereby enabling the pathogen to infect a range of rice varieties [23,24]. Moreover, a proteomic analysis of the xylem sap from tomato plants was previously conducted, resulting in the identification of 14 candidate effector proteins from the infected strain (Fo f.sp. lycopersici; Fol). These proteins are characterized by carrying signal peptides, lacking transmembrane domains, and having relatively small molecular weights (<300 amino acids) [25]. Whole-genome screening of *F. oxysporum* CCS043 was conducted based on the general characteristics of the effector proteins, resulting in the identification of a total of 10 effector proteins.

### 3.3. Root Exudates of Continuously Cultivated R. glutinosa Enhance F. oxysporum CCS043 Infection in the Rhizosphere

The main characteristics of continuously cropped *R. glutinosa* are the low survival rate of its seedlings, a lack of emergence of the seedlings, and no expansion of the roots. Such circumstances may result in a reduction in economic productivity. The occurrence of this phenomenon can be attributed to the comprehensive interplay of various dose–effect relationships in the “soil–plant–microorganism” system. A large number of studies have shown that root exudates will accumulate continuously in the soil, causing harm to the next crop [26], which is an important factor that causes soil-borne diseases and leads to continuous cropping problems. The key components of allelochemicals, including ferulic acid, catalpol, and mullein glycoside, were identified and confirmed in previous studies [10,11]. The concentrations of ferulic acid, catalpol, and mullein glycoside in the rhizosphere soil of continuously cropped *R. glutinosa* have been determined to be 100 μmol/L, 23.1 μg/g, and 12.6 μg/g, respectively [27,28]. To simulate a continuous cropping environment, the physiological characteristics of *F. oxysporum* CCS043 were assessed through the exogenous application of key components of root exudates. It was found that the mycelial growth of *F. oxysporum* CCS043 and the activities of representative fungal infection enzymes are promoted to varying degrees at concentrations of ferulic acid, verbascoside, and catalpol, respectively, of 100 μmol/L, 20 μg/mL, and 20 μg/mL. In addition to inducing proliferation, the promotion of pathogenic fungi by allelochemicals can also lead to changes in the enzyme activities of pectinase and cellulose, thus affecting the pathogenic ability of *F. oxysporum* CCS043 [29,30]. In this study, the greatest promotion of pectin and cellulase activities was observed at a mullein glycoside concentration of 20 μg/mL. With the addition of mullein glycoside, it was speculated that pathogenic bacteria could degrade the host cell wall faster and achieve the purpose of their invasion. In addition, pectinase was the main pathogenic factor of *F. oxysporum* CCS043 whose activity was directly related to the degree of disease of the plants. Similarly, cellulase activity also affected the pathogenicity of *F. oxysporum* CCS043 to a certain extent [31]. The secretion of hydrolase is affected by the carbon source and nitrogen sources present. Allelopathic substances serve as a carbon source for *F. oxysporum* CCS043, thereby promoting the secretion of pectinase and cellulase. In the interaction between host plants and pathogenic microorganisms, the cell-wall-degrading enzymes and toxins produced by the invasion of pathogenic bacteria can promote the rapid invasion of these pathogenic bacteria and their survival and reproduction in the host. Furthermore, the cell wall constitutes the primary line of defense against the invasion of pathogenic bacteria. As a result of accelerated dissolution of the cell wall, the spore vitality and rate of mycelial growth in pathogenic bacteria increase, thereby enhancing their infectivity and colonizing ability. Consequently, pathogenic bacteria become the dominant flora, disrupting the rhizosphere’s microecology and exacerbating disease in *R. glutinosa*. This also contributes to the inability to cultivate it continuously.

### 3.4. F. oxysporum CCS043 Mainly Proliferates and Grows Through Amino Acid Metabolism

In the complex and diverse damage by pathogenic bacteria, it is impossible for them to harm the host plant by a single means. From the transcriptomic analysis, ferulic acid, mullein glycoside, and catalpol were found to increase the proliferation effect of *F. oxysporum* CCS043’s cytokinesis through gene regulation of the amino acid metabolism pathway, as the key components of *R. glutinosa* root exudates. The main components of the cell membrane of *F. oxysporum* CCS043 are proteins and lipids, and the differential expression of metabolic pathway genes means that treatment with the root exudates induced division and growth activity in *F. oxysporum* CCS043, which inevitably led to faster mycelial growth in *F. oxysporum* CCS043.

### 3.5. The Molecular Mechanism of R. glutinosa Root Exudates Promoting F. oxysporum CCS043’s Proliferation

The cell wall of plants is mainly composed of an intercellular layer, a primary wall, and a secondary wall. The main component of the intercellular layer of the cell wall is pectin, which is mainly responsible for cell adhesion and intercellular extrusion. Cellulose represents the primary component of the primary wall and the secondary wall, comprising 25% to 50% of the dry weight of the cell wall. It confers considerable mechanical strength in plant cell walls. Therefore, pectin and cellulase are the two most important cell-wall-degrading enzymes secreted by fungi. Pectinase facilitates the dissolution of the intercellular layer of the host cell wall, severing the intercellular connections in plant tissues, loosening the tissue structure, and facilitate the growth of *F. oxysporum* CCS043’s mycelium between the host cells. Cellulase has the capacity to degrade cellulose into monosaccharides and polysaccharides, thereby destroying the host cell wall and supporting the growth of *F. oxysporum* CCS043 [32,33]. Hydrolase has the potential to assist *F. oxysporum* CCS043 in overcoming the initial physical barrier presented by the host, facilitating the degradation of the cell wall as a source of nutrients, and markedly enhancing the infection capacity of *F. oxysporum* CCS043. In terms of the genes identified in this study, both *GH7* and *CBH2* act as 1,4-*β*-*cellobiosidases* to promote the production of cellulose [32], *CELB* promotes the transfer of cellulase activity [33], the *MCH* gene has previously been identified as a regulator of hydrolase activity [34], *ogl* promotes the catabolism of glucose, and glucose affects the catabolism of cellulase [35]. The significant expression of these genes means that the efficiency of *F. oxysporum* CCS043’s hydrolase secretion becomes higher, with improved infection activity. Additionally, the results of qPCR revealed the significant expression of the effector-protein-coding genes *ChiC*, *VRDA*, *csn*, *chitinase*, and *MUC3_17*. Once a pathogenic effector has stimulated the immune response in a plant, it is essential that the correct number of immune receptors is present and that the response occurs at the correct time and location in order to activate the subsequent immune signal transduction cascade [36]. Significant expression of this effector protein will interfere with the correct timing of the host immune response, which will lead to the aggravation of host immune impairment and the outbreak of continuous cropping problems. The physiological responses of the effector proteins of *F. oxysporum* CCS043 specialized to continuously cropped *R. glutinosa* were measured. The genes *ChiC* and *csn*, which encode effector proteins, are not only highly expressed at the transcriptional level but also caused hypersensitivity reactions in isolated leaves of *R. glutinosa* in vitro, identifying them as core candidate effector proteins in *F. oxysporum* CCS043. In summary, with the addition of ferulic acid, catalpol, and verbascoside, *F. oxysporum* CCS043 significantly increased in its infective and pathogenic capabilities through enhanced cell proliferation, hydrolase activity, and expression of effector-protein-encoding genes, thereby intensifying its harmful effects.

## 4. Materials and Methods

In this experiment, the *R. glutinosa* variety employed was “Wen 85-5”, which is extensively cultivated in Wenxian, Henan, China. The *F. oxysporum* CCS043 strain [37], which is specific to *R. glutinosa*, was isolated from *R. glutinosa* plants stricken by root rot disease. It was sequenced and stored in our laboratory.

### 4.1. Determination of the Physiological Indicators of F. oxysporum CCS043

Pectinase activity determination: A 1 mg/mL galacturonic acid standard solution in sodium acetate buffer (pH 4.8) was prepared. Different volumes (0–0.6 mL) of this solution were mixed with 1.5 mL DNS reagent in test tubes, heated in a boiling water bath for 5 min, cooled to room temperature, diluted to 10 mL, and measured at 540 nm.

Cellulase activity detection: A 1 mg/mL glucose standard solution was prepared. Different volumes (0–0.6 mL) were mixed with 1.5 mL DNS reagent, heated in a boiling water bath for 5 min, cooled, diluted to 10 mL, and measured at 520 nm.

Absolute quantification of *F. oxysporum* CCS043 in the rhizosphere of *R. glutinosa*: Specific primers designed for a plasmid gene fragment using Primer 5.0 (3 to 10^−4^ ng/μL) were used for qRT-PCR with SYBR Green MasterMix (Q712, Vazyme, Nanjing, China). The data were analyzed using BIO-Rad IQ5 v2.1

Detection of ROS in *R. glutinosa* leaves in vitro: The ROS levels in infected leaves were measured using an ELISA kit (YX-031501P, SINOBESTBIO, Shanghai, China) following the manufacturer’s protocol [38].

DAB staining: The infected leaves were stained with 1 mg/mL DAB solution for 12 h in the dark, washed with tap water, decolorized with 96% ethanol, and photographed.

### 4.2. The RNA-Seq Data Analysis

Adapters, low-quality sequences, and reads with over 5% unknown bases (N) were removed using SOAPnuke v1.4.0 [39]. Clean reads were aligned with the reference genome using HISAT2 v2.1.0 [40]. The transcript expression levels were normalized using the FPKM method. Differentially expressed genes (DEGs) were identified using DESeq2 v1.30, with significant DEGs defined by *p* ≤ 0.05 and log2|FoldChange| ≥ 2. Up- and downregulated genes were quantified. A KEGG analysis was performed on the DEGs, and visualizations were generated using OmicShare tools [41].

### 4.3. RT-qPCR Data Analysis

The primer design was conducted using Primer5, with EF-1α selected as the reference gene (F: GCTGGTGACTCCAAGAACGA; R: CATCTTGACGATGGCGGAGT). The Taq Pro Universal SYBR qPCR Master Mix kit (Q712, Vazyme, Nanjing, China) was utilized for the qRT-PCR procedure. The relative gene expression levels between different samples were determined using the 2^−ΔΔCt^ method for data calculation.

### 4.4. Genome Assembly

The *F. oxysporum* CCS043 genome was sequenced using the DNBSEQ platform. Genomic DNA was extracted and randomly fragmented, and fragments of 300–400 bp were recovered through electrophoresis, ligated with adapters, and prepared for sequencing. Low-quality and adapter sequences were filtered to generate high-quality clean data. The assembly involved four steps: (1) self-correction of the subreads using Pbdagcon and Falcon Consensus to generate high-confidence corrected reads; (2) the assembly of corrected reads using tools like Celera and Falcon v0.3.0 (https://github.com/PacificBiosciences/falcon, accessed 24 September 2023) to select the optimal results; (3) single-base correction of the assemblies using GATK v1.6-13; and (4) scaffold construction using Illumina long-fragment data, with the gaps filled using SSPACE Basic v2.0 and PBJelly2 (https://sourceforge.net/projects/pb-jelly/, accessed 10 September 2023).

### 4.5. Phylogenetic Analysis

Based on the alignment results from MUMmer v3.22, the genome sequence of the target strain was reordered according to the genome sequence of the reference strain. The sequences were scaled down proportionally to the total lengths of the two genomes, constructing the X- and Y-axes of a two-dimensional collinearity plot and the upper and lower axes of a linear collinearity plot. Subsequently, BLASTp was used for bidirectional alignment between the target strain protein set P1 and the reference strain protein set P2 (P1 vs. P2 and P2 vs. P1). The best alignment result (best hit) for each protein was selected, and consistent protein pairs from both alignments were retained. The consistency value of these protein pairs was calculated as the average of the consistency values from both alignments. Finally, the protein pairs were scaled down proportionally according to their positional information and plotted onto the graph. Additionally, the gene sets of six *Fusarium oxysporum* strains (*F. oxysporum* Fo5176, *F. oxysporum* FoC.Fus2, *F. oxysporum* Forc016, *F. oxysporum* HDV247, *F. oxysporum* MN25, and *F. oxysporum* CCS043) were clustered using CD-HIT v4.6.6. The final clustered gene set was defined as the pan gene set. Core gene sets were extracted from the sequences present in all of the samples within the clusters, while genes unique to individual samples were classified as specific gene sets. Subsequently, the gene sets were translated into protein sets, and a phylogenetic tree was constructed for the six strains using the PHYML algorithm in TreeBeST v1.9.2.

### 4.6. Screening of the Effector Proteins in F. oxysporum CCS043

The screening of the effector proteins based on the whole-genome sequencing data for *F. oxysporum* CCS043 from the rhizosphere of *R. glutinosa* was conducted in four steps. Initially, the SignalP 5.0 server [42] was used for an online prediction analysis of the N-terminal signal peptides for all of the proteins encoded by the FO genome, resulting in protein sequences with signal peptides. Subsequently, TMHMM v2.0 [43] was utilized to screen for proteins with transmembrane domains, as these proteins may have been membrane receptors, anchoring proteins, or ion channels on the membrane. Continuing with ProtComp v.9.0 [44], subcellular localization prediction was performed on the preliminarily predicted FO protein sequence to screen for proteins that can be secreted outside the cell. Then, the Big-PI predictor [45] (https ://gpcr.biocomp.unibo.it/predgpi/pred.htm, accessed 27 September 2023) was then applied to further identify GPI-anchored proteins. Additionally, CalMolWt (http://www.tofms.org/CalMW/MYMWele.asp, accessed 29 September 2023) was employed to calculate the cysteine content of all of the candidate sequences given that effector proteins typically have a wide range of residue numbers, from 50 to 300 aa, and are cysteine-rich. Finally, the PHIB (http://phi-blast.phi-base.org/, accessed 29 September 2023) database [46] was used for the sequence alignment, from which effector proteins annotated as pathogenic genes and that had higher scores were selected for functional validation.

### 4.7. ORF Cloning of the Effector Factors in Full Length

The correct ORF sequence for the target gene from the genomic results of *F. oxysporum* CCS043 in the rhizosphere of *R. glutinosa* was identified using ORFfinder (https://www.ncbi.nlm.nih.gov/orffinder/, accessed on 6 July 2024). Primers were designed at both ends of this sequence with the aid of the software SnapGene v4.2.4 and CE Design v1.04. Subsequently, PCR amplification was carried out using the Phanta Max Super-Fidelity DNA Polymerase P505-d1 kit (Vazyme, Nanjing, China).

### 4.8. Agrobacterium GV3101 Transformation and Infection

The plasmid transformation was carried out using the freeze–thaw method. In brief, 1 mL of positive bacterial culture was selected and added to 50 mL of LB (containing 50 µg/mL Rif and 100 µg/mL Kan), and the mixture was shaken until it turned orange-yellow. The bacteria were then collected through centrifugation at 5000 rpm for 10 min at 4 °C, after which the supernatant was gently poured off and the remaining liquid was discarded. The bacteria were resuspended in MS liquid medium containing 100 μmol/L acetosyringone and 10 mmol/L MgCl_2_ until the OD600 value reached 1.0, followed by incubation in the dark at 28 °C for 3 h, which prepared them for subsequent infection of the *R. glutinosa* leaves.

### 4.9. In Vitro Infection of the R. glutinosa Leaves 

Tissue culture seedlings of *R. glutinosa*, approximately 45 days old and with uniform growth, were used. The middle leaves were cut on a sterile workbench and placed into an empty tissue culture bottle with MS medium to keep them moist. For Agrobacterium infection, the same points on both sides of the midrib on a leaf’s underside were selected. The Agrobacterium solution was then injected using a syringe without a needle (with three leaves per group, each infected with different strains) and marked accordingly. Post-injection, the leaves were placed onto an agarose plate for daily observation and photography.

### 4.10. Statistical Analysis

Dunnett’s multiple-comparison test was used in GraphPad Prism 9.3.0 software for the analysis of the significance.

## 5. Conclusions

This study confirmed that root exudates from continuous cropping of *R. glutinosa* promote the proliferation of *F. oxysporum* CCS043 in its rhizosphere. Treatments with ferulic acid, verbascoside, and catalpol enhanced the mycelial growth of *F. oxysporum* CCS043, with a significant increase observed after 4 days. The transcriptomic analysis revealed that the root exudates accelerate *F. oxysporum* CCS043’s hyphal growth through amino acid metabolism, enhancing its competitiveness in the soil microbiota, increasing its infectious enzyme activity and effector protein secretion, and boosting its infectivity and pathogenicity. This process exacerbates continuous cropping stress and allows *F. oxysporum* CCS043 to dominate the soil microbiome. The genome analysis identified two key effector proteins, ChiC (GME8876_g) and csn (GME9251_g), which cause hypersensitive reactions in *R. glutinosa* leaves. Fluorescence quantification confirmed that these genes are highly expressed during early transcription, establishing them as core effectors with critical pathogenic roles. This study provides insights into the fungal disease outbreaks caused by microecological imbalances in the rhizosphere, offering a theoretical and technical foundation for addressing obstacles to continuous cropping in *R. glutinosa*.

## Figures and Tables

**Table 1:** Overview of the genome assembly of *F. oxysporum* CCS043 in the rhizosphere of *R. glutinosa*.

Feature	*F. oxysporum* CCS043
Survey-estimated genome size	61.05 Mb
Final assembled genome size	47.67 Mb
Scaffold number and N50	45 (N50:4451.03 Kb)
Repeat size (%)	6.45
GC content (%)	48.31
Gene number	16,423
Average gene length	1615.58
Average CD length	1477.40

**Table 2:** Candidate pathogenic effect factors.

Gene ID	Gene Length	AA Length	Protein Annotation
GME7369_g	705	235	Increased_virulence
GME8855_g	789	263	Plant_avirulence_determinant
GME8876_g	897	299	Plant_avirulence_determinant
GME4726_g	699	233	Plant_avirulence_determinant
GME15395_g	348	116	Increased_virulence
GME5525_g	384	128	Reduced or increased_virulence
GME5646_g	399	133	Reduced or increased_virulence
GME5707_g	807	269	Plant_avirulence_determinant
GME9251_g	867	289	Reduced_virulence
GME10991_g	420	140	Plant_avirulence_determinant

## Data Availability

The data presented in this study are available in article or Supplementary Material here.

## Supplementary Materials

The following are available online at https://www.mdpi.com/article/10.3390/plants14010038/s1. Figure S1: Evaluation of *F. oxysporum* CCS043’s genome using a k-mer analysis. Figure S2: The colony diameter of *F.oxysporum* under the treatment of different root exudates. Figure S3: Number of transmembrane domain proteins. Table S1: Annotation of the entire genome of *F. oxysporum* CCS043 using 15 functional databases. Table S2: Classification and statistics of *F. oxysporum* CCS043 transposons in *R. glutinosa*’s root system. Table S3: Statistics on the non-coding RNAs of *F. oxysporum* CCS043 in *R. glutinosa*’s root system. Table S4: Statistics on the number of genes in 6 strains of *F. oxysporum* CCS043. Table S5: Core–pan gene protein statistics table. Table S6: Quality control filtering of raw transcriptome data. Table S7: Range of amino acid residues. Table S8: Prediction of subcellular localization of proteins with signal peptides. Table S9: GPI-anchored protein prediction using the Big-PI predictor. Table S10: Screening results of effector proteins rich in cysteine residues. Table S11: Screening and classification of differential genes.
